# Predictors of In-Hospital Cardiac Arrest Outcomes: A Single-Center Observational Study

**DOI:** 10.3390/jcm14217868

**Published:** 2025-11-05

**Authors:** Maria Aggou, Barbara Fyntanidou, Andreas S. Papazoglou, Marios G. Bantidos, Nikolaos Vasileiadis, Dimitrios Vasilakos, Haralampos Karvounis, Dimitrios V. Moysidis, Athina Nasoufidou, Panagiotis Stachteas, Paschalis Karakasis, Konstantinos Fortounis, Eleni Argyriadou, Efstratios Karagiannidis, Vasilios Grosomanidis

**Affiliations:** 1 Department of Anesthesiology, AHEPA University Hospital, 54636 Thessaloniki, Greece; mariaangou@auth.gr (M.A.); dvasilak@auth.gr (D.V.); argiriadou@auth.gr (E.A.); vgrosoma@auth.gr (V.G.); 2 Department of Emergency Medicine, AHEPA University Hospital, 54636 Thessaloniki, Greece; fyntanidou@auth.gr (B.F.); mpantidos@auth.gr (M.G.B.); 3 Department of Cardiology, Athens Naval Hospital, 11521 Athens, Greece; 4 Second Department of Cardiology, Hippokration General Hospital, 54642 Thessaloniki, Greece; athinaso@auth.gr (A.N.); panstacha@auth.gr (P.S.); pkarakaa@auth.gr (P.K.); 5 Amedes Medizinische Dienstleistungen GmbH, 37081 Göttingen, Germany; nikolaos.vasileiadis@hotmail.com; 6 First Department of Cardiology, AHEPA University Hospital, 54636 Thessaloniki, Greece; hkarvounis@gmail.com; 7 Medical School, Aristotle University of Thessaloniki, 54124 Thessaloniki, Greece; dimoysidis@gmail.com; 8 Department of Surgery, Papageorgiou General Hospital, 56429 Thessaloniki, Greece; kostfort@yahoo.gr

**Keywords:** in-hospital cardiac arrest, resuscitation, risk stratification, logistic regression, Utstein style

## Abstract

**Background/Objectives:** In-hospital cardiac arrest (IHCA) carries high mortality and substantial risk of neurological and functional impairment. Given that contemporary, clinically relevant risk models remain limited, especially within Southern European systems, the aim of this study was to develop a process-aware model for bedside risk stratification. **Methods:** We retrospectively analyzed a single-center cohort from a prospectively maintained resuscitation registry (AHEPA University General Hospital, Thessaloniki). Adults (≥18 years) with index IHCA in 2017–2019 were included. Utstein-defined variables underwent univariable screening, LASSO selection, and collinearity checks before multivariable logistic regression for in-hospital mortality. We assessed discrimination (AUC) and calibration (Hosmer–Lemeshow). **Results:** Among 826 IHCAs, 137 survived to discharge and 689 died. Higher mortality was independently associated with longer CPR (aOR = 1.115, 95% CI: 1.080–1.158), older age (aOR = 1.034, 95% CI: 1.014–1.055), and CCU location (aOR = 7.303, 95% CI: 2.557–25.798), while operating room (aOR = 0.029, 95% CI: 0.003–0.252), ICU/HDU (aOR = 0.203, 95% CI: 0.065–0.630), and an initial shockable rhythm (aOR = 0.297, 95% CI: 0.144–0.611) were protective. Longer time to CPR initiation also predicted mortality (aOR = 1.746, 95% CI: 1.001–3.162). Model performance was strong (AUC = 0.897, 95% CI: 0.865–0.928) with good calibration (Hosmer–Lemeshow *p* = 0.879). **Conclusions:** A process-aware model integrating patient factors, intra-arrest metrics, and location showed excellent internal performance for predicting IHCA mortality. Findings reaffirm the prognostic importance of age, rhythm, and resuscitation timeliness/intensity and support future work extending prediction to neurological/functional outcomes and testing targeted care bundles in high-risk strata.

## 1. Introduction

In-hospital cardiac arrest (IHCA) constitutes a high-consequence event with increased in-hospital mortality, substantial neurological disability among survivors, and resource-intensive post-resuscitation care [1,2]. The median incidence across hospitals is almost 4 cases per 1000 admissions, with a median survival rate to discharge of ≅20%; nonetheless, the reported survival rates range from 0 to 42% across studies and regions, reflecting marked heterogeneity in the prognostic course [3,4]. This inter-hospital variation highlights the need for fair performance assessment and benchmarking [5]. 

Efforts to harmonize IHCA reporting have improved comparability but remain uneven. Despite international consensus templates (i.e., Utstein-style reporting), large sustained IHCA registries are relatively few and concentrated in selected health systems—most prominently the American Heart Association’s Get With The Guidelines–Resuscitation (GWTG–Resuscitation), the UK–Ireland National Cardiac Arrest Audit (NCAA), and national registries such as Denmark’s DANARREST (Danish In-Hospital Cardiac Arrest Registry) and Sweden’s SRCR (Swedish Registry for Cardiopulmonary Resuscitation) [6,7,8,9,10]. Across these initiatives, case ascertainment, inclusion criteria, variable definitions and completeness, and outcome endpoints (e.g., survival to discharge vs. 30-day survival; neurological metrics) vary considerably. 

Meanwhile, IHCA data collection is inherently challenging, and most large-scale investigations have prioritized incidence, overall survival, and temporal trends rather than a systematic evaluation of prognostic determinants [11]. Outcomes are influenced by both non-modifiable factors (such as age and comorbidities) and modifiable aspects of care and process (including arrest location, monitoring, response delays, and treatment strategies). While many of the modifiable factors represent plausible targets for improvement, they are often inconsistently captured across datasets [12,13,14,15]. 

Given (a) the historical emphasis on clinical audit and institutional-level quality improvement, (b) the frequent restriction to limited variable sets, and (c) the paucity of Southern-European IHCA data, the current study analyzes a contemporary, prospective IHCA cohort in Northern Greece to identify independent predictors of in-hospital mortality using a multivariable framework suited to a large candidate-variable set. The objective is to derive a clinically relevant risk model that supports bedside assessment and guides pragmatic quality-improvement initiatives.

## 2. Materials and Methods

### 2.1. Study Population and Setting

We conducted a single-center observational cohort study using a prospectively maintained in-hospital resuscitation registry at AHEPA University General Hospital, a tertiary academic center in Thessaloniki serving Northern Greece. Eligible participants were adults (≥18 years) who experienced an index IHCA during the same admission and received resuscitation between 2017 and 2019. We excluded cardiac arrests that occurred outside hospital grounds and admissions with a documented do-not-attempt-resuscitation (DNAR) order.

### 2.2. Definitions

Definitions followed Utstein-style recommendations for in-hospital resuscitation [6]. Cardiac arrest was defined as cessation of cardiac mechanical activity with absence of signs of circulation. Initial rhythms were categorized as shockable [ventricular fibrillation (VF) or ventricular tachycardia (VT)] and non-shockable [asystole or pulseless electrical activity (PEA)]. A resuscitation attempt was defined as confirmed arrest with delivery of chest compressions and/or defibrillation. Return of spontaneous circulation (ROSC) denoted restoration of a palpable pulse with an organized rhythm; sustained ROSC was not recorded as a separate endpoint. Time intervals were measured from arrest recognition to the event of interest. 

### 2.3. Data Collection

AHEPA University General Hospital maintains a hospital-wide, prospectively documented resuscitation registry for quality-assurance and performance monitoring. For every IHCA, the responding team completes a standardized record at the time of the event (or immediately thereafter). From 2017–2018, data elements reflected the 2004 Utstein template; in 2019, the registry was updated to the 2019 Utstein template [6,16]. To ensure consistency across forms, records from 2017–2018 (2004 template) were harmonized to the 2019 terminology. The core analytic variables used in this study are equivalent across the 2004 and 2019 templates. Included were patient demographics, arrest location and context, initial rhythm, time intervals [recognition, cardiopulmonary resuscitation (CPR) initiation, defibrillation, ROSC], interventions, and outcomes. 

### 2.4. Ethical Approval

The study protocol was reviewed and approved by the Directory Board and the Scientific Council of AHEPA University General Hospital (approval details: 14969/9 April 2024). As this investigation entailed a retrospective analysis of prospectively documented entries from the hospital resuscitation registry, the requirement for individual informed consent was waived in accordance with national regulations and the Declaration of Helsinki [17]. Data handling complied with institutional policies and applicable data-protection legislation (European Union General Data Protection Regulation, EU GDPR).

### 2.5. Statistical Analysis

Continuous variables are summarized as mean ± standard deviation or median with interquartile range (IQR), and categorical variables as frequencies with percentages (%). Group comparisons were performed using Student’s *t* test or the Mann–Whitney U test for continuous data and chi-squared (or the Fisher’s exact test when expected cell counts were <5) for categorical data. 

To identify candidate predictors for the primary study endpoint (i.e., in-hospital death), we first performed univariable binary logistic regression analyses for each baseline clinical or resuscitation-related characteristic, reporting unadjusted odds ratios (ORs) with 95% confidence intervals (CIs). Variables with *p* < 0.10 were carried forward. Predictors with >20% missing data were excluded, and the remainder were analyzed using a complete-case approach.

Given the relatively large number and overlapping clinical nature of the candidate predictors (n = 83 variables with potential clinical relevance) in relation to the number of observed events (n = 689 deaths), we applied a penalized regression approach to reduce dimensionality and avoid overfitting. Specifically, least absolute shrinkage and selection operator (LASSO) logistic regression was used to identify the most informative and independent predictors for inclusion in the final multivariable model. To further assess collinearity, we calculated variance inflation factors (VIFs), considering VIF > 10 as high and 5–10 as moderate collinearity. Highly or moderately collinear variables were excluded from the multivariable regression model. The final multivariable logistic regression model included predictors selected by LASSO and confirmed by VIF screening. Adjusted ORs (aORs) with 95% CIs and *p*-values are reported. 

The performance of the final multivariable logistic regression model was evaluated in terms of discrimination and calibration. Discrimination was assessed by calculating the area under the receiver operating characteristic (ROC) curve (AUC) with corresponding 95% CIs. An AUC of 0.5 was considered no better than chance, whereas values of 0.7–0.8, 0.8–0.9, and >0.9 were interpreted as acceptable, good, and excellent discrimination, respectively. Calibration was examined using the Hosmer–Lemeshow goodness-of-fit test with 10 risk deciles; a non-significant *p*-value indicated adequate calibration. In addition, calibration plots (observed vs. predicted event rates across deciles of risk) were generated to provide a visual assessment of model fit.

Finally, given the strong adjusted association observed with CCU location, we conducted a setting-based subgroup analysis (CCU vs. non-CCU). We first ran confirmatory multivariable logistic regressions within each subgroup using the independent predictors from the overall model to assess whether effects remained consistent within each setting. Because the CCU subgroup had a reduced effective sample size, we anticipated wider confidence intervals; therefore, we complemented the models with unadjusted two-group comparisons (chi-squared for categorical variables; Mann–Whitney U test for continuous variables), summarized as counts (%) and medians with IQRs. This analysis was descriptive only and did not inform variable selection or multivariable model specification. All analyses were conducted using the R version 4.4.2. All tests were two-sided with α = 0.05, and *p*-values are reported as exact values unless <0.001.

## 3. Results

### 3.1. Population Description

A total of 826 index IHCA events met inclusion criteria; 137 patients survived to discharge and 689 died during hospitalization.

#### 3.1.1. Baseline Characteristics

The cohort was 65% male with a median age of 73 years (IQR 61–80); survivors were younger than non-survivors [64 (56–75) vs. 74 (63–80), *p* < 0.001]. Reasons for admission most commonly included chronic coronary artery disease (21%), acute myocardial infarction (AMI) (16%), and pneumonia/lung pathology (16%). AMI and surgery/trauma were proportionally more frequent among survivors, whereas heart failure as a comorbidity was more common among non-survivors; other recorded comorbidities showed broadly similar distributions among survivors and non-survivors.

#### 3.1.2. Direct Cause at Recognition

At the time of arrest, hypotension and acute respiratory distress syndrome were frequent proximate causes of IHCA and occurred more often in non-survivors, whereas primary cardiac arrhythmia as the direct cause of IHCA was comparatively more common in survivors.

#### 3.1.3. Arrest Location and Timing

Cardiac arrest occurred most often on the ward (34%) and in the CCU (26%), with smaller proportions in the emergency department (ED) (16%), intensive care unit/high-dependency unit (ICU/HDU) (9.9%), catheterization laboratory (6.1%), operating room (3.8%), and dialysis unit (4.1%). Location patterns differed by outcome: ICU/HDU and operating-room arrests were more frequent among survivors, whereas CCU arrests among non-survivors. Arrest timing skewed to afternoon/night shifts (61%), and one quarter occurred on weekends; both timing patterns were more pronounced in non-survivors.

#### 3.1.4. Arrest Characteristics and Interventions

At recognition, 24% presented with an initial shockable rhythm (VF/VT), a finding more common in survivors. Airway management with mechanical ventilation was required in 82% overall but less often among survivors. The median total epinephrine dose administered during resuscitation was 4 milligrams (4 ampules) (IQR 2–6), with lower doses among survivors. Detailed survivor–non-survivor comparisons are provided in Table 1.

### 3.2. Predictors of In-Hospital Mortality

Univariable logistic regression analyses identified 35 variables associated with in-hospital death at *p* < 0.10 (Supplementary Table S1). After excluding five variables with >20% missing data, 30 variables were entered into a LASSO regression with 10-fold cross-validation, which retained 20 variables with non-zero coefficients after collinearity assessment (VIF ≤ 5). These 20 variables were forced into the final multivariable logistic regression model (Table 2; VIFs shown in Supplementary Table S2).

### 3.3. Independent Predictors of In-Hospital Mortality

Based on the final multivariable logistic regression model, several variables were independently associated with in-hospital mortality. Longer CPR duration was associated with higher odds of death (aOR = 1.115, 95% CI: 1.080–1.158, *p* < 0.001). Older age also predicted higher mortality, with a 3% risk increase per year increase (aOR = 1.034, 95% CI: 1.014–1.055, *p* = 0.001). CCU arrests had higher odds of death (aOR = 7.303, 95% CI: 2.557–25.798, *p* < 0.001), whereas operating room (aOR = 0.029, 95% CI: 0.003–0.252, *p* = 0.002) and ICU/HDU arrests (aOR 0.203, 95% CI 0.065–0.630, *p* = 0.006) had lower odds. A shockable initial rhythm decreased the risk of death (aOR = 0.297, 95% CI: 0.144–0.611, *p* = 0.001). Absence of a need for assisted ventilation during CPR was also associated with lower mortality (aOR = 0.026, 95% CI: 0.002–0.393, *p* = 0.009). Time to CPR initiation showed an association with higher mortality (aOR = 1.746, 95% CI: 1.001–3.162, *p* = 0.049). Full adjusted estimates are reported in Table 2. 

### 3.4. Model Performance

The final multivariable logistic regression model demonstrated excellent discrimination for in-hospital mortality, with an AUC of 0.897 (95% CI: 0.865–0.928). Calibration by the Hosmer–Lemeshow test showed no evidence of lack of fit (X^2^ = 3.75, df = 8, *p* = 0.879) suggesting that the predicted probabilities closely matched the observed event rates across risk deciles. Visual inspection of the calibration plot further confirmed good agreement between predicted and observed outcomes.

### 3.5. Additional Subgroup Analysis

In the confirmatory multivariable logistic regressions, the independent predictors of the overall model that persisted differed per subgroup. In CCU, only CPR duration remained independently associated with in-hospital mortality (aOR = 77.8, 95% CI: 2.1–2.8 × 10^6^, *p* < 0.001); estimates for age, initial rhythm, time to CPR initiation, ICU/HDU and operating-room locations were directionally similar but not statistically significant, with wide confidence intervals (Supplementary Table S3). In non-CCU, older age (aOR = 1.03, 95% CI: 1.01–1.06, *p* = 0.001), longer CPR duration (aOR = 2.45, 95% CI: 1.82–3.45, *p* < 0.001), initial shockable rhythm (aOR = 0.29, 95% CI: 0.15–0.55, *p* < 0.001), and operating-room location (aOR = 0.25, 95% CI: 0.06–0.98, *p* = 0.04) remained independent predictors (Supplementary Table S4). In unadjusted comparisons, CCU arrests occurred in older patients (median 75 vs. 71 years, *p* < 0.001), had longer CPR duration (20 vs. 15 min, *p* = 0.049), higher epinephrine doses (4 vs. 3 ampules, *p* = 0.041), and higher in-hospital mortality (90% vs. 81%, *p* = 0.002) than non-CCU arrests. With regard to comorbidities, CCU patients had a heavier chronic disease burden—heart failure (27% vs. 13%, *p* < 0.001), coronary artery disease (29% vs. 15%, *p* < 0.001), and valvular heart disease (5.1% vs. 1.6%, *p* = 0.005) (Supplementary Tables S5 and S6). Rhythm distributions and several pre-arrest care processes also differed between settings.

## 4. Discussion

Contemporary attempts to synthesize IHCA risk stratification models are scarce, particularly outside large national registries, let alone within Southern European healthcare systems. In that direction, our study sought to characterize IHCA events within a tertiary center serving Northern Greece and develop a data-driven prediction model for IHCA in-hospital mortality. The final multivariable model integrated demographic, arrest-related and care-setting variables, and demonstrated strong performance: AUC 0.897 (95% CI 0.865–0.928), with good calibration and a non-significant Hosmer–Lemeshow result, indicating reliable risk discrimination and close agreement between predicted and observed outcomes. These findings support the model’s potential utility in guiding clinical decision-making, prioritizing monitoring strategies, and informing the design of future interventional studies in Southern Europe.

First, our modelling strategy—combining patient factors with intra-arrest processes and granular location—positions this work between lean benchmarking models and more process-rich single-center studies. In contrast, the GWTG-Resuscitation risk-standardization models [risk-standardized survival rate (RSSR); a hospital’s survival rate adjusted for average case-mix], including the original 9-variable version and the 2023 update, as well as the UK NCAA models, were specifically designed to enable fair between-hospital comparisons. They restrict predictors largely to pre-arrest/at-arrest variables and achieve moderate discrimination with excellent calibration; they intentionally omit treatment-dependent intra-arrest metrics to avoid penalizing (or rewarding) hospitals for performance within the very metric being compared [12,13,15]. For GWTG–Resuscitation, the RSSR specifically estimates hospital-level survival to discharge and, in its 2023 update, recalibrated coefficients while retaining the pre-/at-arrest covariate philosophy (e.g., age, illness category, initial rhythm, arrest location, and acuity markers present at arrest). The NCAA models, built for national audit, predict ROSC > 20 min and survival to discharge from variables recorded at team arrival (age, prior length of stay, reason for attendance, location, first documented rhythm), with results issued as risk-adjusted comparative hospital reports rather than bedside predictions. On the other hand, in our study variables like time to CPR, CPR duration, assisted ventilation, and detailed arrest location were included to yield higher discrimination but may limit transportability unless such variables are captured consistently across sites. Practically, the two approaches are complementary: a process-aware local model for bedside risk estimation alongside national frameworks for cross-site benchmarking and surveillance over time.

Our cohort’s baseline profile (older median age, high crude mortality, heavy off-hours burden) is broadly consistent with recent European IHCA reports, but it differs from settings with wider DNAR adoption and higher monitoring intensity, where short-term survival is generally—though not uniformly—higher [18,19,20,21]. Our location mix—substantial ward (34%) and CCU (26%) burden with comparatively fewer ICU/HDU events (9.9%), particularly among non-survivors—parallels some large studies; however, prior multi-hospital cohorts have reported ICU arrest proportions as high as 59% [14,22]. Off-hours disadvantages described internationally aligned with our univariable findings, but the association attenuated after adjustment—suggesting that day–night and weekday–weekend differences possibly operate through contextual factors [22,23].

With respect to predictors retained in our multivariable model, the direction and relative weight of key variables—older age, non-shockable rhythm, and need for assisted ventilation—mirror consensus patterns from previous large studies [14]. Our inclusion of granular arrest locations (ICU/HDU and operating room) reproduces the monitored-area advantage and procedural-area protection previously reported [22,24]. Nonetheless, it is noteworthy that presumed cardiac-arrest cause (e.g., AMI)—highlighted in some prior studies—did not retain significance in our adjusted analysis [14]. We believe this likely reflects not misclassification of presumed etiology but rather the more proximal pathophysiological signal of the initial rhythm—particularly shockability and its treatment responsiveness—which showed a significant association in our model (aOR = 0.297, 95% CI: 0.144–0.611, *p* = 0.001). In parallel, longer CPR duration appears to proxy refractory arrest physiology, while the need for assisted ventilation likely indexes more significant respiratory compromise and greater illness severity at/after the time of arrest and CPR. Timeliness of resuscitation also mattered heavily in our data (longer time to CPR initiation associated with higher mortality), underscoring modifiable levers such as rapid recognition/response, expedited defibrillation for shockable rhythms, and early, standardized airway/ventilation strategies. Collectively, these process-related markers (time to CPR, CPR duration, assisted ventilation) inform both case severity and intra-/post-resuscitation responsiveness, and represent potential targets for intervention. The CCU finding merits closer examination due to its strong association with mortality (aOR = 7.303, 95% CI: 2.557–25.798, *p* < 0.001). In our cohort, CCU arrests occurred in older patients, with longer CPR and higher epinephrine exposure, and had higher mortality than non-CCU arrests despite similar or greater presence of staff and monitoring—supporting a case-mix/context explanation rather than an intrinsic “unit-label” effect. This interpretation is strengthened by more frequent severe comorbidities in the CCU (e.g., heart failure and valvular heart disease; *p* < 0.001 and *p* = 0.005). Additionally, the proportion with a shockable initial rhythm was similarly low in CCU and non-CCU groups (26% vs. 23%, *p* = 0.353), consistent with a population enriched for non-coronary pathology. Notably, definitions of the modern CCU have evolved toward the “cardiac ICU (CICU)”, a unit commonly managing decompensated heart failure, respiratory failure, cardiogenic/mixed shock, and other non-AMI and non-coronary critical illnesses. In this clinical milieu, the low prevalence of shockable rhythms in our CCU population is expected and likely reflects higher acuity and disease complexity [25,26].

Collectively, the above support the credibility and clinical relevance of our findings, while acknowledging certain constraints. Strengths include a prospectively maintained, hospital-wide registry with Utstein harmonization, a transparent modelling pipeline that screened a broad candidate set, and excellent internal performance with strong discrimination and supportive calibration, with explicit checks for collinearity. Alignment with the 2019 in-hospital Utstein template further supports comparability with contemporary cohorts. Limitations include the single-center setting (local organizational features may limit generalizability) and the absence of certain clinically pertinent measures. In particular, prospective assessment of frailty with dedicated indices (e.g., Clinical Frailty Scale or baseline functional status) would be informative, and standardized neurological outcomes beyond survival (e.g., CPC or mRS at discharge and follow-up) were not recorded. Moreover, specialized procedure-level data from different departments—such as revascularization rates in AMI and damage-control/emergent surgery protocols—were not systematically available, precluding adjusted analyses of their effect. These limitations do not detract from the principal signals but define clear priorities for subsequent work.

A logical next step would be to extend outcome assessment beyond survival. Standardized neuroprognostication could be achieved by prospectively recording the Cerebral Performance Category (CPC) and/or the modified Rankin Scale (mRS) at discharge and at later time points (e.g., 90 days) [27,28,29]. The model would also benefit from external (geographic and temporal) validation—with light recalibration if needed—and pragmatic integration into routine clinical workflows to confirm transportability and clinical impact. For interventional work, our findings support testing targeted care bundles in high-risk strata (e.g., CICU patients flagged by the model)—combining enhanced monitoring with predefined escalation pathways, standardized early airway/ventilation strategies, and a consistent post-arrest bundle—while explicitly evaluating feasibility and resource aspects.

## 5. Conclusions

We developed a clinically oriented prediction model for in-hospital mortality after IHCA that demonstrates strong performance and provides clear guidance for bedside decision-making. By clarifying which variables matter most, the model can help target monitoring, intervention, and post-arrest care more efficiently. Future work should validate these findings in diverse settings, incorporate standardized neurological and functional outcomes, and determine whether model-guided pathways translate into improved patient-centered results.

## Figures and Tables

**Table 1:** Baseline demographic and clinical characteristics.

Variable	Total CA Population (n = 826)	Survivors (n = 137)	Non-Survivors (n = 689)	*p*-Value
Male gender	540 (65%)	100 (73%)	440 (64%)	0.051
Age (median [Q1, Q3])	73 [61, 80]	64 [56, 75]	74 [63, 80]	<0.001
**Reason for admission**				
Cancer	43 (5.2%)	7 (5.1%)	36 (5.2%)	>0.999
Surgery or trauma	49 (5.9%)	16 (12%)	33 (4.8%)	0.004
Stroke or central nervous system pathology	38 (4.6%)	4 (2.9%)	34 (4.9%)	0.421
Pneumonia or lung pathology	136 (16%)	21 (15%)	115 (17%)	0.790
Aortic dissection	22 (2.7%)	2 (1.5%)	20 (2.9%)	0.504
Heart failure or pulmonary edema	106 (13%)	13 (9.5%)	93 (13%)	0.254
Cardiac arrhythmia	31 (3.8%)	2 (1.5%)	29 (4.2%)	0.194
Coma	21 (2.5%)	3 (2.2%)	18 (2.6%)	>0.999
Syncope	15 (1.8%)	1 (0.7%)	14 (2.0%)	0.489
Chronic kidney disease	35 (4.2%)	5 (3.6%)	30 (4.4%)	0.887
Chronic coronary artery disease	177 (21%)	35 (26%)	142 (21%)	0.241
Acute myocardial infarction	129 (16%)	32 (23%)	97 (14%)	0.009
Gastrointestinal pathology	49 (5.9%)	4 (2.9%)	45 (6.5%)	0.151
**Direct cause of IHCA**				
Hypotension	232 (30%)	21 (16%)	211 (33%)	<0.001
Sepsis	13 (1.7%)	1 (0.8%)	12 (1.9%)	0.604
Cardiac arrhythmia	160 (21%)	55 (42%)	105 (16%)	<0.001
Acute respiratory distress syndrome	277 (36%)	30 (23%)	247 (39%)	0.001
**Patient comorbidities**				
Mental illness	15 (1.8%)	2 (1.5%)	13 (1.9%)	>0.999
Hypertension	21 (2.5%)	5 (3.6%)	16 (2.3%)	0.546
Coronary artery disease	156 (19%)	32 (23%)	124 (18%)	0.179
Diabetes mellitus	10 (1.2%)	4 (2.9%)	6 (0.9%)	0.115
Lung pathology	35 (4.2%)	6 (4.4%)	29 (4.2%)	>0.999
Stroke or central nervous system pathology	23 (2.8%)	3 (2.2%)	20 (2.9%)	0.858
Chronic kidney disease	42 (5.1%)	3 (2.2%)	39 (5.7%)	0.140
Cancer	57 (6.9%)	7 (5.1%)	50 (7.3%)	0.471
Heart failure	136 (16%)	13 (9.5%)	123 (18%)	0.022
Valvular heart disease	21 (2.5%)	5 (3.6%)	16 (2.3%)	0.546
Previous cardiac arrest	6 (0.8%)	2 (1.7%)	4 (0.7%)	0.574
**Cardiac arrest rhythm**				
Initial shockable rhythm (VF/VT)	196 (24%)	68 (50%)	128 (19%)	<0.001
**Interventions at time of arrest**				
Mechanical ventilation required for airway management	650 (82%)	93 (71%)	557 (84%)	<0.001
Total epinephrine dose (median [Q1, Q3])	4.00 [2.00, 6.00]	2.00 [1.00, 3.00]	4.00 [2.00, 6.00]	<0.001
**Cardiac arrest location**				
Operating room	31 (3.8%)	15 (11%)	16 (2.3%)	<0.001
Emergency department	132 (16%)	19 (14%)	113 (16%)	0.541
ICU/HDU	82 (9.9%)	22 (16%)	60 (8.7%)	0.013
Ward	282 (34%)	47 (34%)	235 (34%)	>0.999
CCU	215 (26%)	21 (15%)	194 (28%)	0.003
Catheterization laboratory	50 (6.1%)	9 (6.6%)	41 (6.0%)	0.935
Dialysis unit	34 (4.1%)	4 (2.9%)	30 (4.4%)	0.592
**Timing of cardiac arrest**				
Weekend arrest	200 (24%)	21 (15%)	179 (26%)	0.011
Afternoon or night shift	505 (61%)	66 (48%)	439 (64%)	0.001

**Table 2:** Multivariable regression outcomes.

Variable	aOR	95% CI	*p*-Value
Age	1.034	1.014–1.055	<0.001
Acute myocardial infarction (as the reason for admission)	0.424	0.141–1.321	0.130
Diabetes mellitus (as comorbidity)	0.196	0.030–1.787	0.108
Hypotension (as direct cause of IHCA)	1.728	0.870–3.566	0.127
Heart failure (as comorbidity)	1.833	0.728–5.322	0.227
Cardiac arrest in the operating room	0.029	0.003–0.252	0.002
Cardiac arrest in the CCU	7.303	2.557–25.798	<0.001
Cardiac arrest in the ICU/HDU	0.203	0.065–0.630	0.006
Shift at time of arrest	1.279	0.901–1.829	0.172
Total number of ALS procedures performed	1.554	0.448–5.738	0.495
Resuscitation team activated	0.641	0.164–2.383	0.511
Time to CPR initiation	1.746	1.001–3.162	0.049
CPR duration	1.115	1.080–1.158	<0.001
Time to first epinephrine dose	0.904	0.750–1.143	0.326
CPR performed by department staff and resuscitation team	0.500	0.151–1.404	0.217
No assisted ventilation required for airway management	0.026	0.002–0.393	0.009
Mechanical ventilation used for airway control (during CPR)	1.154	0.437–2.841	0.763
Initial shockable rhythm (VF/VT)	0.297	0.144–0.611	<0.001
Physician present during the cardiac arrest	1.306	0.548–3.325	0.559
Nurse participation during CPR	0.180	0.014–1.706	0.154

## Data Availability

Study data will be available upon reasonable request from the corresponding study authors (A.S.P. and E.K.).

## Supplementary Materials

The following supporting information can be downloaded at: https://www.mdpi.com/article/10.3390/jcm14217868/s1, Table S1. Univariable logistic regression outcomes, Table S2. Variance inflation factor values of the variables included in the multivariable logistic regression model, Table S3. CCU multivariable logistic regression outcomes, Table S4. Non-CCU multivariable logistic regression outcomes, Table S5. Chi-squared comparisons between CCU and non-CCU cardiac arrest location, Table S6. Wilcoxon test comparisons between CCU and non-CCU cardiac arrest location.

## Abbreviations

The following abbreviations are used in this manuscript: ALS: advanced life support; AMI: acute myocardial infarction; aOR: adjusted odds ratio; AUC: area under the receiver operating characteristic curve; CCU: coronary care unit; CICU: cardiac intensive care unit; CI: confidence interval; CPC: Cerebral Performance Category; CPR: cardiopulmonary resuscitation; DANARREST: Danish In-Hospital Cardiac Arrest Registry; df: degrees of freedom; DNAR: do not attempt resuscitation; ED: emergency department; EU: European Union; GDPR: General Data Protection Regulation; GWTG–Resuscitation: Get With The Guidelines–Resuscitation; HDU: high-dependency unit; ICU: intensive care unit; IHCA: in-hospital cardiac arrest; IQR: interquartile range; LASSO: least absolute shrinkage and selection operator; mRS: modified Rankin Scale; NCAA: National Cardiac Arrest Audit; OR: odds ratio; PEA: pulseless electrical activity; ROC: receiver operating characteristic; ROSC: return of spontaneous circulation; RSSR: risk-standardized survival rate; SRCR: Swedish Registry for Cardiopulmonary Resuscitation; UK: United Kingdom; VF: ventricular fibrillation; VIF: variance inflation factor; VT: ventricular tachycardia.
