# Supplementary material for: Predictors of In-Hospital Cardiac Arrest Outcomes: A Single-Center Observational Study
Source: J Clin Med. 2025 Nov 5;14(21):7868. doi: 10.3390/jcm14217868 (PMC12608470; doi:10.3390/jcm14217868)
Supplement: Supplementary file 1 [file jcm-14-07868-s001.zip › jcm-3957296-supplementary.pdf]

Table S1. Univariable logistic regression outcomes.

Variable	OR	95%CI	p-value
Gender	0.654	0.430-0.975	0.041
Age	1.028	1.016-1.040	<0.001
**Reason for admission**			
Cancer	1.024	0.473-2.556	0.956
Surgery or trauma	0.38	0.206-0.729	0.003
Stroke or central nervous system pathology	1.726	0.674-5.853	0.310
Pneumonia or lung pathology	1.107	0.679-1.878	0.695
Aortic dissection	2.018	0.580-12.734	0.348
Heart failure or pulmonary edema	1.488	0.835-2.864	0.203
Cardiac arrhythmia	2.966	0.880-18.488	0.140
Coma	1.198	0.399-5.166	0.774
Syncope	2.821	0.560-51.331	0.318
Chronic kidney disease	1.202	0.498-3.577	0.709
Chronic coronary artery disease	0.757	0.498-1.170	0.199
Acute myocardial infarction	0.538	0.346-0.853	0.007
Gastrointestinal pathology	2.323	0.924-7.807	0.112
**Direct cause**			
Hypotension	2.553	1.586-4.292	<0.001
Sepsis	2.465	0.479-45.106	0.388
Cardiac arrhythmia	0.268	0.178-0.402	<0.001
Acute respiratory distress syndrome	2.095	1.368-3.293	0.001
**Patient comorbidities**			
Mental illness	1.298	0.353-8.363	0.733
Hypertension	0.628	0.241-1.946	0.371
Coronary artery disease	0.72	0.468-1.132	0.144
Diabetes mellitus	0.292	0.082-1.156	0.059
Lung pathology	0.959	0.417-2.601	0.928
Stroke or central nervous system pathology	1.335	0.450-5.724	0.644
Chronic kidney disease	2.68	0.954-11.199	0.104
Cancer	1.453	0.687-3.577	0.368
Heart failure	2.073	1.173-3.963	0.018
Valvular heart disease	0.628	0.241-1.946	0.371
Previous cardiac arrest	0.391	0.075-2.846	0.282
**Cardiac arrest location**			
Operating room	0.193	0.093-0.405	<0.001

Emergency department	1.218	0.736-2.114	0.461
ICU/HDU	0.499	0.298-0.860	0.010
Ward	0.991	0.677-1.467	0.964
CCU	2.165	1.348-3.634	0.002
Catheterization laboratory	0.9	0.446-2.017	0.782
Dialysis unit	1.514	0.585-5.159	0.443
Advanced department	0.285	0.172-0.479	<0.001
**Timing of cardiac arrest**			
Weekend arrest	1.939	1.205-3.259	0.009
Shift at time of arrest	1.303	1.057-1.612	0.014
Days of hospitalization before arrest	1.006	0.993-1.023	0.402
**ALS procedures present before arrest**			
All ALS procedures	0.615	0.368-1.063	0.070
Intravenous line and monitor	0.736	0.506-1.064	0.105
Intravenous line	0.778	0.262-1.866	0.608
No ALS procedures	1.286	0.536-3.815	0.608
New monitoring initiated	0.797	0.548-1.154	0.233
**Resuscitation team activation and arrival**			
Resuscitation team activated	1.543	1.044-2.263	0.028
Time from recognition to resuscitation team call	1.064	0.653-1.817	0.810
Time from resuscitation team call to arrival	1.284	1.012-1.639	0.042
Arrest occurred after resuscitation team arrival	1.551	0.816-3.270	0.210
Resuscitation team present during arrest	1.482	0.778-3.128	0.263
Arrest confirmation time by resuscitation team	1.75	1.232-2.578	0.003
**CPR**			
Time to CPR initiation	1.848	1.314-2.669	0.001
Chest compressions	1.233	0.635-2.308	0.522
CPR duration	1.127	1.099-1.158	<0.001
Time to first defibrillation	1.412	1.059-1.907	0.021
Time to first epinephrine dose administered	1.198	1.035-1.400	0.019
Total epinephrine dose (ampules)	1.51	1.336-1.730	<0.001
Administration of amiodarone	1.014	1.000-1.0360	0.079
CPR performed by department staff and resuscitation team	1.285	0.975-1.668	0.066
**Airway and ventilation**			
Time to airway establishment	1.247	1.053-1.482	<0.001
No assisted ventilation required for airway management	0.033	0.007-0.100	<0.001
Mechanical ventilation used for airway control (during CPR)	2.107	1.360-3.221	0.001

Supraglottic airway or bag-mask ventilation used	0.923	0.551-1.615	0.768
Tracheostomy	0.985	0.319-4.295	0.981
**Rhythm**			
Initial rhythm	0.453	0.364-0.561	<0.001
Shockable rhythm (VF/VT)	0.232	0.157-0.340	<0.001
**Witnesses/ Bystanders**			
Recognition	0.922	0.341-2.110	0.860
Witnesses	0.162	0.009-0.763	0.074
Accompanying person	1.072	0.658-1.821	0.787
Resuscitation team	1.102	0.570-2.347	0.786
Physician and nurse	0.761	0.489-1.157	0.212
Physician	0.612	0.422-0.890	0.010
**Nurse participation**			
Nurse participation during CPR	2.391	0.905-5.690	0.059
Nurse starting CPR	1.042	0.661-1.687	0.864
**ALS/ILS training**			
ALS trained physician	1.534	0.340-5.106	0.521
ILS trained nurse	1.125	0.754-1.670	0.560
**Other**			
Emergency call	0.718	0.168-2.128	0.597
Crash cart	2.088	0.843-4.723	0.090

*Abbreviations: ALS: advanced life support; CCU: coronary care unit; CI: confidence interval; CPR: cardiopulmonary resuscitation; HDU: high-dependency unit; ICU: intensive care unit; OR: odds ratio; VF: ventricular fibrillation; VT: ventricular tachycardia.

Table S2. Variance inflation factor values of the variables included in the multivariable logistic regression model.

**Variable**	**VIF**
Age	1.108
Acute myocardial infarction (as the reason for admission)	1.409
Diabetes mellitus (as comorbidity)	1.060
Hypotension (as direct cause)	1.122
Heart failure (as comorbidity)	1.146
Cardiac arrest in the operating room	2.291
Cardiac arrest in the CCU	1.274
Cardiac arrest in the ICU/HDU	2.064
Shift at time of arrest	1.137
Total number of ALS procedures performed	2.205

Resuscitation team activated	3.921
Time to CPR initiation	1.388
CPR duration	1.142
Time to first epinephrine dose	1.469
CPR performed by department staff and/or resuscitation team	2.885
No assisted ventilation required for airway management	1.298
Mechanical ventilation used for airway control (during CPR)	1.293
Initial shockable rhythm (VF/VT)	1.217
Physician present during the cardiac arrest	1.986
Nurse participation during CPR	1.465

*Abbreviations: ALS: advanced life support; CCU: coronary care unit; CPR: cardiopulmonary resuscitation; HDU: high-dependency unit; ICU: intensive care unit; VF: ventricular fibrillation; VIF: variance inflation factor; VT: ventricular tachycardia.

Table S3. CCU multivariable logistic regression outcomes.

**Variable**	**aOR**	**95% CI**	**p-value**
Age	1.07	0.98-1.24	0.10
CPR duration	77.8	$2.1–2.8\times10^{6}$	<0.001
Initial shockable rhythm (VF/VT)	0.37	0.007–7.1	0.46
Time to CPR initiation	0.42	0.000009–74.0	0.66

*Other locations as variables are not applicable since the analysis is limited to the CCU. Abbreviations: aOR: adjusted odds ratio; CCU: coronary care unit; CI: confidence interval; CPR: cardiopulmonary resuscitation, VF: ventricular fibrillation; VT: ventricular tachycardia.

Table S4. Non-CCU multivariable logistic regression outcomes.

**Variable**	**aOR**	**95% CI**	**p-value**
Age	1.03	1.01–1.06	0.001
CPR duration	2.45	1.82–3.45	<0.001
Initial shockable rhythm (VF/VT)	0.29	0.15–0.55	<0.001
Time to CPR initiation	1.04	0.53–2.04	0.92
Cardiac arrest in the ICU/HDU	0.60	0.27–1.35	0.21
Cardiac arrest in the operating room	0.25	0.06–0.98	0.04

*Abbreviations: aOR: adjusted odds ratio; CCU: coronary care unit; CI: confidence interval; CPR: cardiopulmonary resuscitation; HDU: high-dependency unit; ICU: intensive care unit; VF: ventricular fibrillation; VT: ventricular tachycardia.

Table S5. Chi-squared comparisons between CCU and non-CCU cardiac arrest location.

Variable / Level	CCU (n=215)	Non-CCU (n=611)	p-value
**No assisted ventilation required for airway management**			0.059
No	199 (96%)	578 (98%)	
Yes	9 (4.3%)	10 (1.7%)	
**Mechanical ventilation used for airway control (during CPR)**			<0.001
No	61 (29%)	85 (14%)	
Yes	147 (71%)	503 (86%)	
**Death**			0.002
No	21 (9.8%)	116 (19%)	
Yes	194 (90%)	495 (81%)	
**Physician present during the cardiac arrest**			0.622
No	143 (67%)	395 (65%)	
Yes	72 (33%)	216 (35%)	
**Nurse participation during CPR**			0.010
No	1 (0.5%)	23 (4.4%)	
Yes	197 (99%)	504 (96%)	
**Previous physician training in life support**			>0.999
ILS/BLS trained physician	3 (1.5%)	10 (2.0%)	
ALS trained physician	197 (99%)	500 (98%)	
**Recognition**			0.148

No	8 (5.1%)	35 (8.7%)	
Yes	149 (95%)	366 (91%)	
**Initial rhythm**			0.033
Asystole	90 (42%)	218 (36%)	
PEA	69 (32%)	253 (41%)	
VF	42 (20%)	119 (19%)	
VT	14 (6.5%)	21 (3.4%)	
**Shockable rhythm (VF/VT)**			0.353
No	159 (74%)	471 (77%)	
Yes	56 (26%)	140 (23%)	
**Resuscitation team present during arrest**			0.375
No	197 (92%)	547 (90%)	
Yes	18 (8.4%)	64 (10%)	
**Hypotension (as direct cause of IHCA)**			0.208
No	156 (73%)	382 (69%)	
Yes	57 (27%)	175 (31%)	
**Sepsis (as direct cause of IHCA)**			0.025
No	213 (100%)	544 (98%)	
Yes	0 (0%)	13 (2.3%)	
**Myocardial infarction (as direct cause of IHCA)**			<0.001
No	174 (82%)	508 (91%)	
Yes	39 (18%)	49 (8.8%)	

**Cardiac arrhythmia (as direct cause of IHCA)**			0.586
No	166 (78%)	444 (80%)	
Yes	47 (22%)	113 (20%)	
**Acute respiratory distress syndrome (as direct cause of IHCA)**			0.266
No	143 (67%)	350 (63%)	
Yes	70 (33%)	207 (37%)	
**Intravenous line and monitor (present before arrest)**			<0.001
No	9 (4.2%)	387 (63%)	
Yes	206 (96%)	224 (37%)	
**Intravenous line (present before arrest)**			<0.001
No	0 (0%)	37 (6.1%)	
Yes	215 (100%)	574 (94%)	
**All ALS procedures (present before arrest)**			<0.001
No	206 (96%)	530 (87%)	
Yes	9 (4.2%)	81 (13%)	
**No ALS procedures (present before arrest)**			<0.001
No	215 (100%)	574 (94%)	
Yes	0 (0%)	37 (6.1%)	
**Witnesses**			0.003
No	1 (0.5%)	30 (4.9%)	
Yes	214 (100%)	581 (95%)	
**Accompanying person (witnesses/ bystanders)**			<0.001

No	209 (97%)	484 (79%)	
Yes	6 (2.8%)	127 (21%)	
**Resuscitation team (witnesses/ bystanders)**			0.787
No	199 (93%)	562 (92%)	
Yes	16 (7.4%)	49 (8.0%)	
**Physician and nurse (witnesses/ bystanders)**			<0.001
No	23 (11%)	206 (34%)	
Yes	192 (89%)	405 (66%)	
**Arrest occurred after resuscitation team arrival**			0.282
No	197 (92%)	544 (89%)	
Yes	18 (8.4%)	67 (11%)	
**Shift at time of arrest**			<0.001
Morning shift	76 (35%)	245 (40%)	
Night shift	68 (32%)	114 (19%)	
Afternoon shift	71 (33%)	252 (41%)	
**Mental illness (patient comorbidities)**			0.377
No	213 (99%)	598 (98%)	
Yes	2 (0.9%)	13 (2.1%)	
**Hypertension (patient comorbidities)**			0.081
No	213 (99%)	592 (97%)	
Yes	2 (0.9%)	19 (3.1%)	
**Coronary artery disease (patient comorbidities)**			<0.001

No	152 (71%)	518 (85%)	
Yes	63 (29%)	93 (15%)	
**Diabetes mellitus (patient comorbidities)**			0.468
No	214 (100%)	602 (99%)	
Yes	1 (0.5%)	9 (1.5%)	
**Lung pathology (patient comorbidities)**			0.662
No	207 (96%)	584 (96%)	
Yes	8 (3.7%)	27 (4.4%)	
**Stroke or central nervous system pathology (patient comorbidities)**			0.150
No	212 (99%)	591 (97%)	
Yes	3 (1.4%)	20 (3.3%)	
**Chronic kidney disease (patient comorbidities)**			0.486
No	206 (96%)	578 (95%)	
Yes	9 (4.2%)	33 (5.4%)	
**Cancer (patient comorbidities)**			0.032
No	207 (96%)	562 (92%)	
Yes	8 (3.7%)	49 (8.0%)	
**Heart failure (patient comorbidities)**			<0.001
No	157 (73%)	533 (87%)	
Yes	58 (27%)	78 (13%)	
**Valvular heart disease (patient comorbidities)**			0.005
No	204 (95%)	601 (98%)	

Yes	11 (5.1%)	10 (1.6%)	
**Cancer (reason for admission)**			0.001
No	213 (99%)	570 (93%)	
Yes	2 (0.9%)	41 (6.7%)	
**Surgery or trauma (reason for admission)**			<0.001
No	214 (100%)	563 (92%)	
Yes	1 (0.5%)	48 (7.9%)	
**Valvular heart disease or endocarditis (reason for admission)**			0.004
No	204 (95%)	538 (88%)	
Yes	11 (5.1%)	73 (12%)	
**Stroke or central nervous system pathology (reason for admission)**			<0.001
No	214 (100%)	574 (94%)	
Yes	1 (0.5%)	37 (6.1%)	
**Hematological pathology (reason for admission)**			0.017
No	215 (100%)	595 (97%)	
Yes	0 (0%)	16 (2.6%)	
**Pneumonia or lung pathology (reason for admission)**			<0.001
No	198 (92%)	492 (81%)	
Yes	17 (7.9%)	119 (19%)	
**Aortic dissection (reason for admission)**			0.020
No	214 (100%)	590 (97%)	
Yes	1 (0.5%)	21 (3.4%)	

**Heart failure or pulmonary edema (reason for admission)**			<0.001
No	152 (71%)	568 (93%)	
Yes	63 (29%)	43 (7.0%)	
**Cardiac arrhythmia (reason for admission)**			<0.001
No	198 (92%)	597 (98%)	
Yes	17 (7.9%)	14 (2.3%)	
**Coma (reason for admission)**			0.024
No	214 (100%)	591 (97%)	
Yes	1 (0.5%)	20 (3.3%)	
**Syncope (reason for admission)**			>0.999
No	211 (98%)	600 (98%)	
Yes	4 (1.9%)	11 (1.8%)	
**Chronic kidney disease (reason for admission)**			0.044
No	211 (98%)	580 (95%)	
Yes	4 (1.9%)	31 (5.1%)	
**Chronic coronary artery disease (reason for admission)**			<0.001
No	127 (59%)	522 (85%)	
Yes	88 (41%)	89 (15%)	
**Acute myocardial infarction (reason for admission)**			<0.001
No	143 (67%)	554 (91%)	
Yes	72 (33%)	57 (9.3%)	
**Sex**			0.941

female	74 (34%)	212 (35%)	
male	141 (66%)	399 (65%)	
**Asystole**			0.107
No	125 (58%)	393 (64%)	
Yes	90 (42%)	218 (36%)	
**PEA**			0.016
No	146 (68%)	358 (59%)	
Yes	69 (32%)	253 (41%)	
**VF**			0.985
No	173 (80%)	492 (81%)	
Yes	42 (20%)	119 (19%)	
**VT**			0.054
No	201 (93%)	590 (97%)	
Yes	14 (6.5%)	21 (3.4%)	

*Abbreviations: ALS: Advanced Life Support; BLS: Basic Life Support; CCU: Coronary Care Unit; CPR: Cardiopulmonary Resuscitation; ILS: Immediate Life Support; PEA: Pulseless Electrical Activity; VF: Ventricular Fibrillation; VT: Ventricular Tachycardia.

Table S6. Wilcoxon test comparisons between CCU and non-CCU cardiac arrest location.

**Variable**	**CCU (median value [Q1-Q3])**	**Non-CCU (median value [Q1-Q3])**	**P (Wilcoxon)**	**Rank-biserial r (95%CI)**
Age (years)	75.0 (67.0–81.0)	71.0 (59.0–79.0)	<0.001	-0.18 (-0.27 to -0.09)

Days of hospitalization before arrest	2.0 (1.0–7.0)	2.0 (0.0–8.0)	0.077	-0.08 (-0.17 to 0.01)
Time from resuscitation team call to arrival (minutes)	2.0 (2.0–3.0)	2.0 (2.0–3.0)	0.774	-0.01 (-0.12 to 0.09)
Time to first defibrillation (minutes)	1.0 (0.0–1.0)	1.0 (1.0–2.0)	0.015	0.21 (0.04 to 0.38)
Time to airway establishment (minutes)	3.0 (2.0–4.0)	3.0 (2.0–4.0)	0.784	-0.01 (-0.12 to 0.09)
CPR duration (minutes)	20.0 (10.0–30.0)	15.0 (5.0–30.0)	0.049	-0.09 (-0.18 to 0.00)
Total epinephrine dose (ampules)	4.0 (2.0–6.0)	3.0 (2.0–6.0)	0.041	-0.10 (-0.19 to -0.00)
Administration of amiodarone (milligrams)	300.0 (300.0–300.0)	300.0 (300.0–300.0)	0.060	0.20 (-0.18 to 0.52)

*Abbreviations: CCU: coronary care unit; CI: confidence interval; CPR: cardiopulmonary resuscitation.
